# Neutralization of SARS-CoV-2 Omicron subvariant BA.2.87.1

**DOI:** 10.1016/j.vaccine.2024.03.007

**Published:** 2024-03-07

**Authors:** Ninaad Lasrado, Annika Rössler, Marjorie Rowe, Ai-ris Y. Collier, Dan H. Barouch

**Affiliations:** Beth Israel Deaconess Medical Center, Harvard Medical School, Boston, MA, USA

**Keywords:** SARS-CoV-2, Omicron, BA.2.87.1, Neutralizing antibody, mRNA

## Abstract

A new highly mutated Omicron subvariant BA.2.87.1 has recently been identified with over 30 amino acid mutations in the Spike protein compared with BA.2, BA.5, XBB.1.5, and JN.1 variants. Mutiple mutations in BA.2.87.1 are located in the N-terminal domain (NTD) rather than in the receptor binding domain (RBD) of the Spike protein. We evaluated neutralizing antibody (NAb) responses to BA.2.87.1 because of its highly mutated sequence and its unique NTD region. Our data show that NAb responses to BA.2.87.1 were lower than to BA.2 but higher than to JN.1, suggesting that BA.2.87.1 is not a further antibody escape variant compared with other currently circulating variants. Moreover, XBB.1.5 mRNA boosting increased NAb titers to all variants tested including BA.2.87.1.

## 1. Introduction

The continued evolution of Severe Acute Respiratory Syndrome Coronavirus 2 (SARS-CoV-2) has resulted in the emergence of highly mutated variants that may evade neutralizing antibodies (NAbs) induced by prior infection and vaccination. The highly mutated Omicron BA.2 subvariant BA.2.86 emerged in Fall 2023, but BA.2.86 did not evade NAbs more effectively than other circulating variants such as XBB.1.5 and EG.5.1 [1]. Moreover, current Coronavirus Disease-19 (COVID-19) XBB.1.5 messenger RNA (mRNA) boosters have been shown to increase serum NAb titers to all these circulating variants [2–4] as well as to enhance clinical effectiveness [5].

**Fig. 1.**
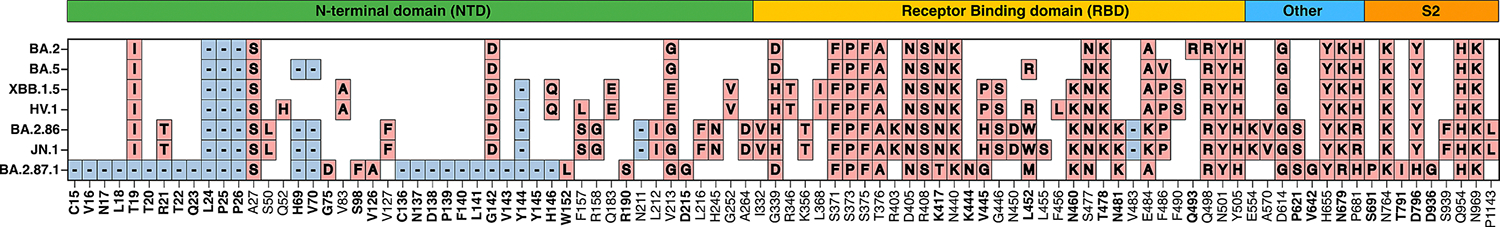
Spike mutations in BA.2.87.1 and current circulating SARS-CoV-2 variants. Spike mutations in BA.2.87.1 and other SARS-CoV-2 variants. Substitutions in the BA.2, BA.5, XBB.1.5, HV.1, BA.2.86, JN.1, and BA.2.87.1 SARS-CoV-2 variants relative to the Wuhan/WIV04/ reference strain (https://gisaid.org/WIV04/) are shown. Amino acid substitutions are indicated in red tiles, and deletions in blue tiles. Substitutions found in BA.2.87.1 relative to BA.2 Omicron variants are highlighted in bold.

A new highly mutated Omicron subvariant BA.2.87.1 has recently been identified with over 100 mutations, including more than 30 amino acid mutations in the Spike protein compared with BA.2, BA.5, XBB.1.5, and JN.1. Many novel mutations in BA.2.87.1 are located in the N-terminal domain (NTD) rather than in the receptor binding domain (RBD) of the Spike protein (Fig. 1). BA.2.87.1 has only been identified in 9 sequences from South Africa to date (Table 1). We evaluated NAb responses to BA.2.87.1 because of its highly mutated sequence and its unique NTD region.

## 2. Results and discussion

**Fig. 2.**
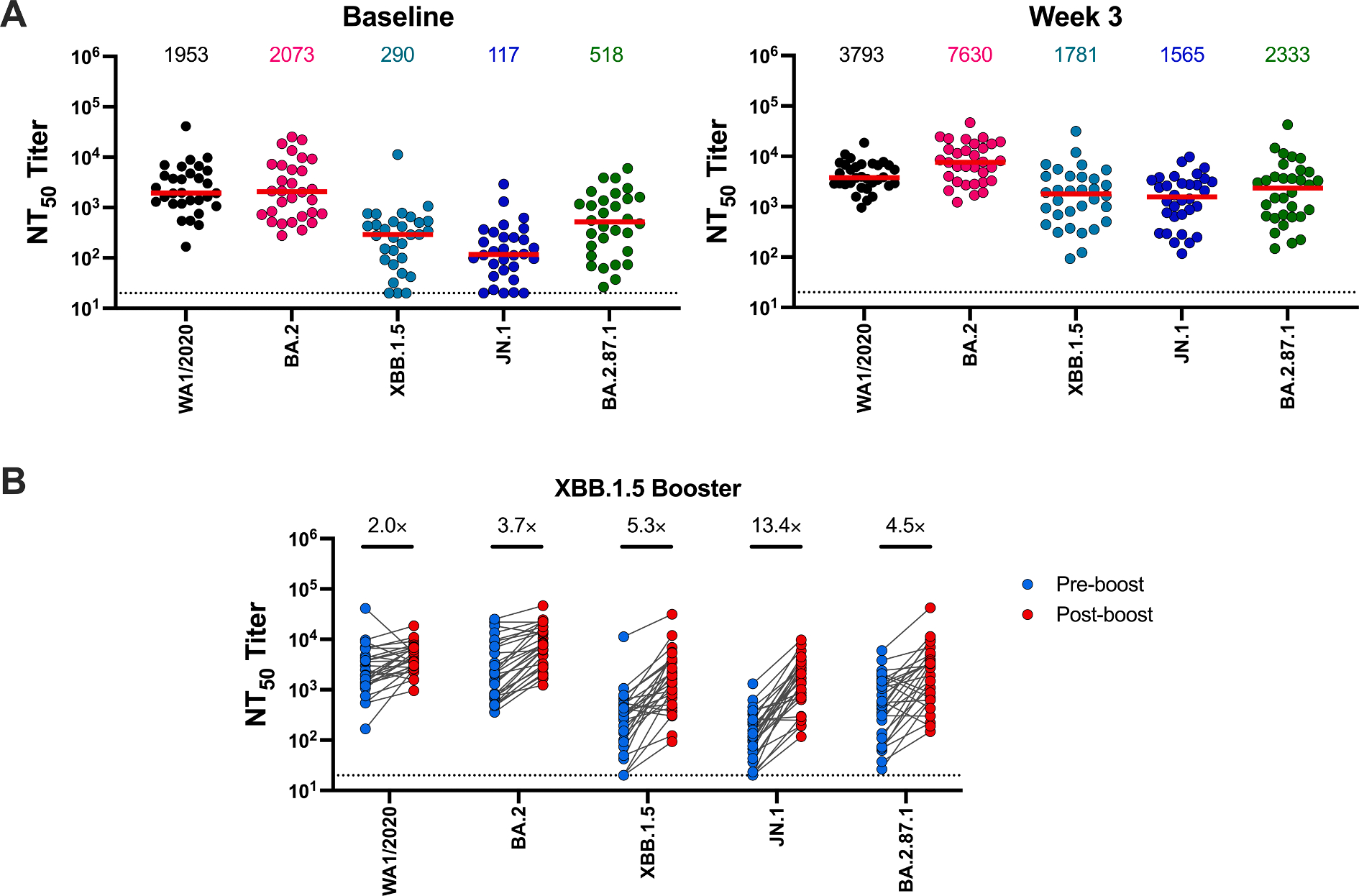
Neutralization of SARS-CoV-2 variants following XBB.1.5 mRNA boosting. (A) Neutralizing antibody (NAb) responses against the WA1/2020, BA.2, XBB.1.5, JN.1, and BA.2.87.1 variants by luciferase-based pseudovirus neutralization assays at baseline and at 3 weeks post-boost in 34 individuals who received an XBB.1.5 mRNA booster in Fall 2023. The horizontal red bar reflects median values. Dotted lines reflect limits of quantitation. (B) Longitudinal paired analysis of serum NAb titers against SARS-CoV-2 WA1/2020, BA.2, XBB.1.5, JN.1, and BA.2.87.1 variants before and after XBB.1.5 mRNA boosting by luciferase-based pseudovirus neutralization assays. Each individual dot represents a single participant (blue, baseline; red, week 3). Fold change increases in NAb titers are depicted numerically. Dotted lines reflect limits of quantitation.

**Fig. 3.**
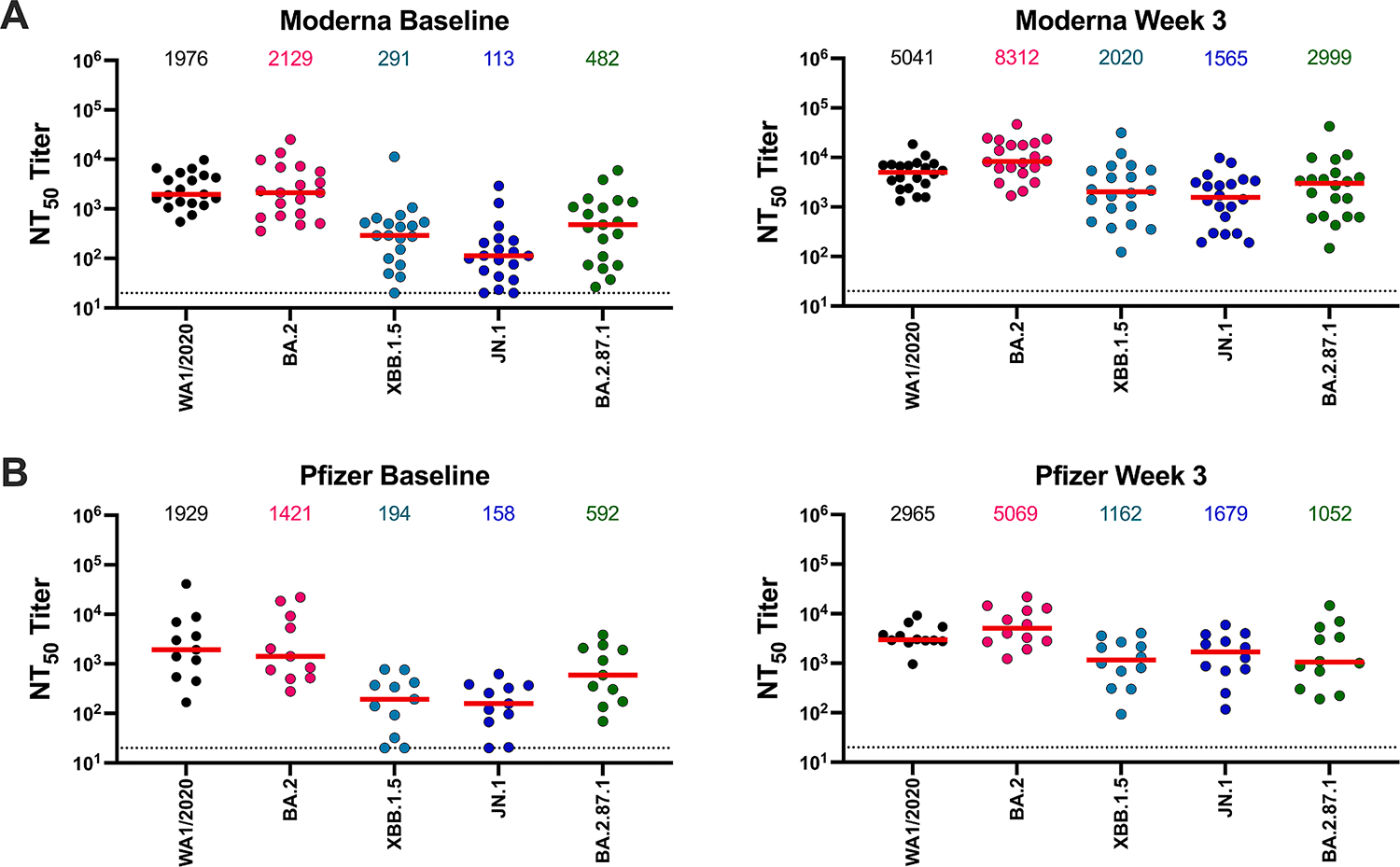
Neutralization of SARS-CoV-2 variants following Moderna or Pfizer XBB.1.5 mRNA boosting. (A) Neutralizing antibody (NAb) responses against the WA1/2020, BA.2, XBB.1.5, JN.1, and BA.2.87.1 variants by luciferase-based pseudovirus neutralization assays at baseline and at 3 weeks post-boost in 21 individuals who received the Moderna XBB.1.5 mRNA booster in Fall 2023. The horizontal red bar reflects median values. Dotted lines reflect limits of quantitation. (B) NAb responses against the WA1/2020, BA.2, XBB.1.5, JN.1, and BA.2.87.1 variants by luciferase-based pseudovirus neutralization assays at baseline and at 3 weeks post-boost in 13 individuals who received the Pfizer XBB.1.5 mRNA booster in Fall 2023. The horizontal red bar reflects median values. Dotted lines reflect limits of quantitation.

We evaluated NAb responses in 34 individuals both before and after XBB.1.5 mRNA boosting (Table 2). Participants had a median of 4 COVID-19 vaccine doses prior to the XBB.1.5 mRNA boost, and 71 % of the participants had at least one documented SARS-CoV-2 infection, although this might be an underestimate of true rate of infections. Baseline NAb responses prior to boosting against WA1/2020, BA.2, XBB.1.5, JN.1, and BA.2.87.1 were 1953, 2073, 290, 117, and 518, respectively (Fig. 2A). NAb responses to all variants increased substantially 3 weeks after the boost (Fig. 2A) to 3793, 7630, 1781, 1565, and 2333, respectively, indicating a 2.0-, 3.7-, 5.3-, 13.4-, and 4.5-fold increase in NAb titers from baseline, respectively (Fig. 2B). Both the Moderna and Pfizer XBB.1.5 mRNA boosters increased NAb titers to these variants (Fig. 3).

Our data show that baseline NAb responses to BA.2.87.1 were 4.0-fold lower than to BA.2 but 4.4-fold higher than to JN.1 prior to XBB.1.5 mRNA boosting. Following XBB.1.5 boosting, NAb responses to BA.2.87.1 were 3.3-fold lower than to BA.2 but 1.5-fold higher than to JN.1. These findings suggest that BA.2.87.1 is not a further antibody escape variant compared with other currently circulating variants such as JN.1. The impact of the numerous NTD mutations remains to be determined, and NAb responses to all current variants including BA.2.87.1 increased following XBB.1.5 mRNA boosting. In contrast with our findings, another study has recently shown that NAb titers to BA.2.87.1 were lower than to XBB.1.5 and JN.1 in China [6], potentially reflecting different SARS-CoV-2 infection and vaccination histories in different geographic regions. Given the continued evolution of SARS-CoV-2, continued surveillance is essential, and future vaccines will need to protect against viruses of increasing diversity [7,8].

## 3. Materials and methods

### 3.1. Study population

A specimen biorepository at Beth Israel Deaconess Medical Center (BIDMC) obtained peripheral blood from individuals in the Fall of 2023 who received a monovalent XBB.1.5 mRNA booster. The BIDMC institutional review board approved this study (2020P000361). All participants provided informed consent. This study included 34 individuals who received an XBB.1.5 mRNA booster (Pfizer-BioNTech or Moderna). Participants were excluded from the immunologic assays if they had a recent history of SARS-CoV-2 infection within 2 weeks of enrollment or if they received immunosuppressive medications.

### 3.2. Pseudovirus neutralizing antibody assay

Neutralizing antibody (NAb) titers against SARS-CoV-2 variants were determined using pseudo-typed viruses expressing a luciferase reporter gene. The pcDNA3.1-BA.2.87.1 SΔCT plasmid used in this study was constructed using BioXP 3250 (TelesisBio). In brief, a luciferase reporter plasmid pLenti-CMV Puro-Luc (Addgene), packaging construct psPAX2 (AIDS Resource and Reagent Program), and Spike protein expressing pcDNA3.1-SARS-CoV-2 SΔCT were co-transfected into human embryonic kidney (HEK)293 T cells (ATCC CRL_3216) with lipofectamine 2000 (ThermoFisher Scientific). Pseudo-typed viruses of SARS-CoV-2 variants were generated using the Spike protein from WA1/2020 (Wuhan/WIV04/2019, GISAID accession ID: EPI_ISL_402124), BA.2 (GISAID ID: EPI_ISL_6795834.2), XBB.1.5 (GISAID ID: EPI_ISL_16418320), JN.1 (GISAID ID: EPI_ISL_18680594), and BA.2.87.1 (GISAID ID: EPI_ISL_18849986). 48 h post-transfection, the supernatants containing the pseudo-typed viruses were collected and purified by filtration with 0.45-μm filter. To determine NAb titers in human sera, HEK293T-hACE2 cells were seeded in 96-well tissue culture plates at a density of 2 × 10^4^ cells per well overnight. Three-fold serial dilutions of heat-inactivated serum samples were prepared and mixed with 60 μl of pseudovirus, and incubated at 37 °C for 1 h before adding to HEK293T-hACE2 cells. 48 h later, cells were lysed in Steady-Glo Luciferase Assay (Promega) according to the manufacturer’s instructions. SARS-CoV-2 neutralization titers were defined as the sample dilution at which a 50 % reduction (NT_50_) in relative light units was observed relative to the average of the virus control wells. NT_50_ titers were calculated by non-linear regression using GraphPad Prism 10 (GraphPad Software, Inc., La Jolla, CA, USA). Titers greater than 1:20 were considered positive.

### 3.3. SARS-CoV-2 variant mutation graph

Amino acid differences in Spike of BA.2.87.1 compared to BA.2, BA.5, XBB.1.5, HV.1, BA.2.86, and JN.1 were analyzed through a Jupyter Notebook from Jesse Bloom (https://github.com/jbloom/SARS2-clade-spike-diffs), and the data were graphed in Adobe Illustrator.

## Figures and Tables

**Table 1:** BA.2.87.1 sequences available in GISAID as of February 20, 2024

Strain	GISAID accession	Date of collection	Continent/Country/Province

hCoV-19/SouthAfrica/NICD-N56614/2023	EPI_ISL_18849985	9/20/23	Africa/South Africa/Gauteng
hCoV-19/SouthAfrica/NICD-N56836/2023	EPI_ISL_18849986	10/7/23	Africa/South Africa/Gauteng
hCoV-19/SouthAfrica/NICD-N57440/2023	EPI_ISL_18849990	10/21/23	Africa/South Africa/Gauteng
hCoV-19/SouthAfrica/NICD-N57176/2023	EPI_ISL_18849987	11/2/23	Africa/South Africa/Limpopo
hCoV-19/SouthAfrica/NICD-N57216/2023	EPI_ISL_18849989	11/12/23	Africa/South Africa/Limpopo
hCoV-19/SouthAfrica/NICD-N57208/2023	EPI_ISL_18849988	11/13/23	Africa/South Africa/Gauteng
hCoV-19/SouthAfrica/NICD-N57469/2023	EPI_ISL_18849991	11/21/23	Africa/South Africa/Limpopo
hCoV-19/SouthAfrica/NICD-R13200/2023	EPI_ISL_18849984	11/30/23	Africa/South Africa/Gauteng
hCoV-19/SouthAfrica/NICD-R13515/2023	EPI_ISL_18845398	12/12/23	Africa/South Africa/Mpumalanga

**Table 2:** Study population.

	All Participants	Pfizer XBB.1.5	Moderna XBB.1.5 N = 21
	N = 34	N = 13	
		

**Age** (years), median (range)	49 (26–73)	51 (30–63)	49 (26–73)
**Sex at birth**, Female	23 (68)	8 (62)	15 (71)
*Race*			
White	30 (88)	11 (85)	19 (90)
Asian	3 (9)	1 (8)	2 (10)
Multiple Races	1 (3)	1 (8)	0
*Ethnicity*			
Non-Hispanic or Latino	33 (97)	12 (92)	21 (100)
Hispanic or Latino	1 (3)	1 (8)	0
*Medical condition*			
Obesity (BMI ≥ 30 kg/m^2^)	5 (15)	4 (31)	1 (5)
Hypertension	7 (21)	3 (23)	4 (19)
Asthma	5 (15)	2 (15)	3 (14)
Lactating	1 (3)	1 (8)	0
*XBB.1.5 mRNA booster*			
Pfizer XBB.1.5 booster	13 (38)	13 (100)	N/A
Moderna XBB.1.5 booster	21 (62)	N/A	21 (100)
*Prior COVID-19 vaccine history*			
BNT (2 doses)/BNT BA.5 (1 dose)	1 (3)	0	1 (5)
BNT (2 doses)/1273 (1 dose)/BNT BA.5 (1 dose)	1 (3)	0	1 (5)
BNT (2 doses)/1273 (2 doses)/1273 BA.5 (1 dose)	1 (3)	1 (8)	0
BNT (2 doses)/Ad26 (1 dose)	1 (3)	1 (8)	0
BNT (2 doses)/Ad26 (1 dose)/BNT (1 dose)	1 (3)	1 (8)	0
BNT (2 doses)/Ad26 (1 dose)/BNT BA.5 (1 dose)	1 (3)	1 (8)	0
BNT (2 doses)/Ad26 (1 dose)/BNT (1 dose)/BNT BA.5 (1 dose)	2 (6)	0	2 (10)
BNT (2 doses)/Ad26 (1 dose)/1273 (1 dose)/BNT BA.5 (1 dose)	2 (6)	0	2 (1)
BNT (3 doses)	1 (3)	0	1 (5)
BNT (3 doses)/BNT BA.5 (1 dose)	2 (6)	1 (8)	1 (5)
BNT (3 doses)/1273 BA.5 (1 dose)	1 (3)	0	1 (5)
BNT (3 doses)/Ad26 (1 dose)/1273 BA.5 (1 dose)	1 (3)	0	1 (5)
BNT (4 doses)/BNT BA.5 (1 dose)	3 (9)	2 (15)	1 (5)
BNT (4 doses)/1273 BA.5 (1 dose)	1 (3)	1 (8)	0
1273 (3 doses)/1273 BA.5 (1 dose)	4 (12)	2 (15)	2 (10)
1273 (3 doses)/BNT BA.5 (1 dose)	1 (3)	1 (8)	0
1273 (4 doses)/1273 BA.5 (1 dose)	2 (6)	0	2 (10)
Ad26 (1 dose)/1273 (2 doses)/1273 BA.5 (1 dose)	1 (3)	0	1 (5)
Ad26 (2 doses)/BNT BA.5 (1 dose)	1 (3)	0	1 (5)
NVX (2 doses)/BNT (1 dose)/BNT BA.5 (1 dose)	2 (6)	1 (8)	1 (5)
NVX (2 doses)/Ad26 (1 dose)/BNT (1 dose)/1273 BA.5 (1 dose)	1 (3)	0	1 (5)
NVX (2 doses)/1273 (3 doses)/1273 BA.5 (1 dose)	1 (3)	0	1 (5)
NVX (3 doses)/BNT BA.5 (1 dose)	1 (3)	0	1 (5)
NVX (3 doses)/1273 BA.5 (1 dose)	1 (3)	1 (8)	0
**Days from prior vaccine to baseline sample**	348 (321–365)	354 (333–368)	336 (320–359)
**Days from XBB.1.5 vaccine to peak sample**	21 (16–29)	24 (19–38)	20 (17–26)
**Days from baseline to peak sample**	42 (29–51)	50 (37–55)	41 (29–48)
**Known COVID-19 positive test** *	24 (71)	11 (85)	13 (62)
1 prior infection	19 (56)	8 (62)	11 (52)
2 prior infections	5 (15)	3 (23)	2 (10)
**Days from most recent positive test to peak sample** **	478 (266–544)	496 (311–567)	466 (268–514)

BNT = BNT162b2 Pfizer COVID-19 mRNA vaccine; 1273 = mRNA-1273 Moderna COVID-19 mRNA vaccine; Ad26 = Ad26.COV2.S Janssen COVID-19 viral-vector vaccine; NVX = Novavax COVID-19 vaccine (protein adjuvanted).

Data displayed as median (range or interquartile range, IQR) and n (%); BMI, body mass index; lactating designation reflects time of last vaccine dose and/or time of sampling. All individuals with known prior infection had mild disease.

* Reported for only those with known prior infection.

** Only reported for participants who contributed a peak sample.

## Data Availability

Data will be made available on request.
